# Impact of the factor V Leiden mutation on the outcome of pneumococcal pneumonia: a controlled laboratory study

**DOI:** 10.1186/cc9213

**Published:** 2010-08-03

**Authors:** Marcel Schouten, Cornelis van't Veer, Joris JTH Roelofs, Marcel Levi, Tom van der Poll

**Affiliations:** 1 Center for Experimental and Molecular Medicine (CEMM), Academic Medical Center, University of Amsterdam, room G2-130, Meibergdreef 9, 1105 AZ, Amsterdam, the Netherlands; 2 Center for Infection and Immunity Amsterdam (CINIMA), Academic Medical Center, University of Amsterdam, room G2-130, Meibergdreef 9, 1105 AZ, Amsterdam, the Netherlands; 3 Department of Pathology, Academic Medical Center, University of Amsterdam, room M2-130, Meibergdreef 9, 1105 AZ, Amsterdam, the Netherlands; 4 Department of Internal Medicine, Academic Medical Center, University of Amsterdam, room F4-119, Meibergdreef 9, 1105 AZ, Amsterdam, the Netherlands

## Abstract

**Introduction:**

*Streptococcus (S.) pneumoniae *is the most common cause of community-acquired pneumonia. The factor V Leiden (FVL) mutation results in resistance of activated FV to inactivation by activated protein C and thereby in a prothrombotic phenotype. Human heterozygous FVL carriers have been reported to be relatively protected against sepsis-related mortality. We here determined the effect of the FVL mutation on coagulation, inflammation, bacterial outgrowth and outcome in murine pneumococcal pneumonia.

**Methods:**

Wild-type mice and mice heterozygous or homozygous for the FVL mutation were infected intranasally with 2*10^6 ^colony forming units of viable *S. pneumoniae*. Mice were euthanized after 24 or 48 hours or observed in a survival study. In separate experiments mice were treated with ceftriaxone intraperitoneally 24 hours after infection and euthanized after 48 hours or observed in a survival study.

**Results:**

The FVL mutation had no consistent effect on activation of coagulation in either the presence or absence of ceftriaxone therapy, as reflected by comparable lung and plasma levels of thrombin-antithrombin complexes and fibrin degradation products. Moreover, the FVL mutation had no effect on lung histopathology, neutrophil influx, cytokine and chemokine levels or bacterial outgrowth. Remarkably, homozygous FVL mice were strongly protected against death due to pneumococcal pneumonia when treated with ceftriaxone, which was associated with more pronounced FXIII depletion; this protective effect was not observed in the absence of antibiotic therapy.

**Conclusions:**

Homozygosity for the FVL mutation protects against lethality due to pneumococcal pneumonia in mice treated with antibiotics.

## Introduction

*Streptococcus pneumoniae *is the leading causative pathogen in community-acquired pneumonia (CAP) [1]. An estimated 570,000 cases of pneumococcal pneumonia occur in the USA annually, resulting in 175,000 hospitalizations. CAP is a frequent cause of sepsis: in a recent sepsis trial 35.6% of the patients suffered from severe CAP, with *S. pneumoniae *as the most frequent cause [2,3]. Worldwide *S. pneumoniae *is responsible for an estimated 10 million deaths annually, making pneumococcal pneumonia and sepsis a major health threat [4]. This together with an increasing incidence of antibiotic resistance in this pathogen [1], urges us to expand our knowledge of the host defense mechanisms that influence the outcome of pneumococcal pneumonia and sepsis.

Severe infection and inflammation have been closely linked to the activation of coagulation and downregulation of anticoagulant mechanisms and fibrinolysis (reviewed in [5]). These hemostatic changes, favoring a procoagulant state, have also been shown in the pulmonary compartment of patients and experimental animals with pneumococcal pneumonia and sepsis [6-10]. The factor V Leiden (FVL) mutation, a missense mutation in the FV gene that replaces arginine at position 506 with glutamine, resulting in resistance of activated FV (FVa) to inactivation by activated protein C (APC) [11], is a major risk factor for venous thrombo-embolism [12]. The high prevalence of this mutation - 4 to 6% in Causasians - despite its prothrombotic effects, has prompted speculation that the mutation might be subject to positive selection pressure during evolution [13]. It has been speculated that FVL carriers might benefit from reduced blood loss during infancy and that the heterozygous FVL carrier status might improve embryo implantation via an unknown mechanism [14,15]. An alternative hypothesis for a survival advantage for heterozygous FVL carriers has been suggested by the observation in patients with severe sepsis that heterozygous FVL carriers had a lower mortality than non-carriers and by animal studies showing an increased survival for heterozygous FVL mice in murine endotoxemia as compared with wild-type (WT) mice [16,17]. However, FVL Leiden mice displayed an unaltered mortality in experimental sepsis induced by viable *Escherichia coli *or group A streptococci [18,19].

The impact of the FVL mutation on the outcome of severe pneumococcal pneumonia has not been studied to date. Therefore, we here investigated whether carriership of the FVL mutation influences the host response to respiratory tract infection by *S. pneumoniae*. For this we infected heterozygous and homozygous FVL mice with viable *S. pneumoniae *and compared their responses with regard to activation of coagulation, inflammation, bacterial outgrowth and dissemination and mortality with those in normal WT mice, both in an untreated and in an antibiotic-treated setting, the latter model more closely mimicking the clinical situation.

## Materials and methods

### Animals

FVL mice carrying an R504Q amino acid mutation [20] were backcrossed four times to a C57BL/6J background (N4) whereafter N4 heterozygous FVL mice were intercrossed to obtain WT, heterozygous and homozygous offspring (confirmed by genotyping) for experiments. Mice were bred and maintained in the animal care facility of the Academic Medical Center, University of Amsterdam, the Netherlands, according to institutional guidelines with free access to food and water. Sex- and age-matched (9 to 11 week old) mice were used in experiments. All experiments were approved by the Institutional Animal Care and Use Committee of the Academic Medical Center.

### Experimental infection and treatment

Pneumonia was induced by intranasal inoculation with about 2 × 10^6 ^colony forming units (CFU) of *S. pneumoniae *serotype 3 (American Type Culture Collection, ATCC 6303, Rockville, MD, USA) as described previously [7,10,21]. Mice were sacrificed after 24 or 48 hours or observed in a survival study. In separate experiments mice were treated once intraperitoneally with ceftraxione (500 μg; Pharmachemie BV, Haarlem, the Netherlands) at 24 hours after infection and were either sacrificed at 48 hours after infection or observed in a survival study. Sample harvesting and processing, and determination of bacterial loads and cell counts were performed as described [7,10,21].

### Assays

Thrombin-antithrombin complexes (TATc; Behringwerke AG, Marburg, Germany), fibrin degradation products (FDP) [22], plasminogen activator inhibitor-1 (PAI-1) [23], keratinocyte-derived chemokine (KC), macrophage inflammatory protein (MIP)-2 (both R&D systems, Minneapolis, MN, USA) and myeloperoxidase (MPO; HyCult Biotechnology, Uden, the Netherlands) were measured by ELISA. Plasminogen activator activity (PAA) was determined by an amidolytic assay [24]. TNF-α, IL-6, monocyte chemoattractant protein (MCP)-1, IL-12p70, interferon (IFN)-γ and IL-10 were measured by cytometric bead array multiplex assay (BD Biosciences, San Jose, CA, USA).

### Factor XIII blots

Immunopurified sheep-anti-human FXIIIA immunoglobulin (Ig) G was obtained from Kordia (Leiden, The Netherlands). For western blotting plasma proteins were separated by polyacrylamide SDS gel electrophoreses under non-reducing conditions and transferred to Immobilon P (Pharmacia, Piscataway, NJ, USA) polyvinylidene difluoride membranes. Membranes were blocked in block buffer containing 5% nonfat dry milk proteins and 0.1% Tween-20 in 50 mM Tris, 150 mM NaCl, pH 7.4 (TBS-T), washed with 0.1% Tween in TBS and incubated overnight with primary antibody in block buffer at 4°C. After washing with 0.1% Tween-20 in TBS membranes were probed with peroxidase labeled secondary antibody for one hour at room temperature in 1% BSA in TBS-T. After washing with TBS-T membranes were incubated with Lumi-LightPlus Western Blotting Substrate (Roche, Mijdrecht, the Netherlands) and positive bands were detected using a Fujifilm LAS-3000 Imager (Fujifilm, Tokyo, Japan). Intensity of the bands was quantified using the AIDA Biopackage 1 D Quantification software (Raytest, Pittsburgh, PA, USA) and was corrected for total protein amount.

### Histology and immunohistochemistry

Paraffin lung sections were stained with H&E or fluorescein isothiocyanate-labeled anti-mouse Ly-6G mAb (Pharmingen, San Diego, CA, USA) as described [25,26]. Fibrin(ogen) staining was performed as described [7,27]. To score lung inflammation, the lung surface was analyzed with respect to the following parameters by a pathologist who was blinded for groups: bronchitis, interstitial inflammation, edema, endothelialitis, pleuritis and thrombus formation. Each parameter was graded on a scale of 0 to 4 (0: absent, 1: mild, 2: moderate, 3: severe, 4: very severe). The total histopathological score was expressed as the sum of the scores for the different parameters. Ly-6G and fibrin(ogen) stained slides were photographed with a microscope equipped with a digital camera (Leica CTR500, Leica Microsystems, Wetzlar, Germany). Stained areas were analysed with Image Pro Plus (Media Cybernetics, Bethesda, MD, USA) and expressed as percentage of the total surface area. The average of 10 pictures was used for analysis.

### Statistical analysis

Data are expressed as box-and-whisker diagrams depicting the smallest observation, lower quartile, median, upper quartile and largest observation or as survival curves. Differences between groups were determined with Kruskal-Wallis - followed by Dunn's multiple comparison test in case of statistical significance, Mann-Whitney *U *test or log rank test where appropriate. Analyses were performed using GraphPad Prism version 4.0 (GraphPad Software, San Diego, CA, USA). *P*-values of less than 0.05 were considered statistically significant.

## Results

### Untreated setting

#### Activation of coagulation

**Figure 1:**
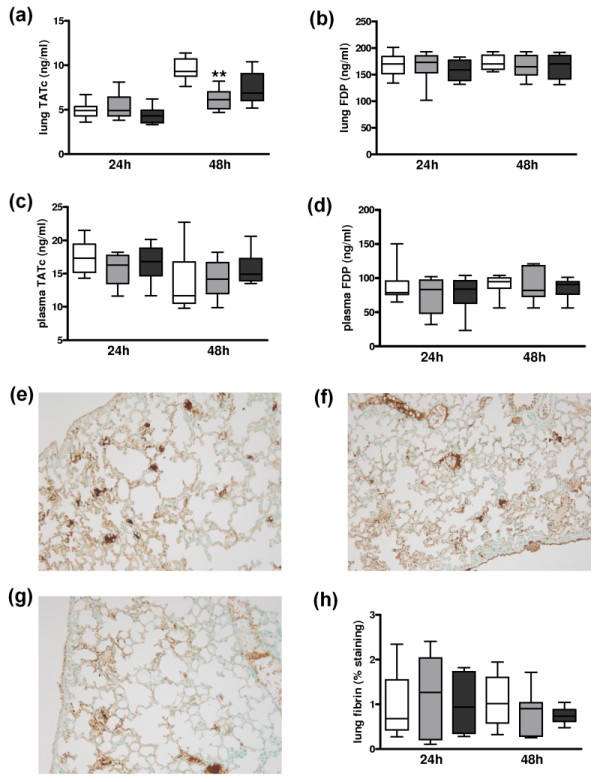
**Activation of coagulation and pulmonary fibrin deposition in untreated pneumococcal pneumonia**. Levels of **(a, c) **thrombin-antithrombin complexes (TATc) and **(b, d) **fibrin degradation products (FDP) in **(a, b) **lung and **(c, d) **plasma 24 and 48 hours after induction of pneumococcal pneumonia in wild-type mice (white, *n *= 8) and mice heterozygous (light grey white, *n *= 8) or homozygous (dark grey white, *n *= 8) for the factor V Leiden mutation. Representative slides of lung fibrin staining (brown) 48 hours after induction of pneumococcal pneumonia in **(e) **wild-type mice, **(f) **mice heterozygous and **(g) **mice homozygous for the factor V Leiden mutation (original magnification × 100). **(h) **Quantitation of pulmonary fibrin content 24 and 48 hours after induction of pneumococcal pneumonia in wild-type mice (white white, *n *= 8) and mice heterozygous (light grey white, *n *= 8) or homozygous (dark grey white, *n *= 8) for the factor V Leiden mutation. Data are expressed as box-and-whisker diagrams depicting the smallest observation, lower quartile, median, upper quartile and largest observation. ** indicates statistical significance as compared to wild-type mice (*P *< 0.01 Kruskal-Wallis test followed by Dunn's multiple comparison test).

To determine whether the FVL mutation impacts on local or systemic activation of coagulation in pneumococcal pneumonia we determined levels of TATc and FDP in lung homogenates (Figures 1a and 1b) and plasma (Figures 1c and 1d) in WT, heterozygous and homozygous FVL mice 24 and 48 hours after intranasal inoculation with viable *S. pneumoniae*. Pulmonary TATc levels were upregulated as compared with baseline (not shown) but were not different between the groups at 24 hours after infection. Remarkably, heterozygous FVL mice had slightly, but statistically significantly, lower pulmonary TATc levels as compared with WT mice after 48 hours, whereas homozygous FVL mice had unaltered TATc levels. There were no differences between the groups in TATc levels in plasma and in FDP levels in lungs or plasma at both time points. To further substantiate activation of coagulation during pneumococcal pneumonia, we performed fibrin(ogen) staining on lungs harvested 24 hours (not shown) or 48 hours after infection (Figures 1e, f and 1g). No differences were seen between WT, heterozygous and homozygous FVL mice (Figure 1h). To determine the impact of the FVL mutation on fibrinolysis we determined PAI-1 levels and PAA in lungs and plasma. There were no differences in PAI-1 and PAA in lungs and plasma at either 24 or 48 hours after infection (data not shown).

#### Pulmonary and systemic inflammation

**Figure 2:**
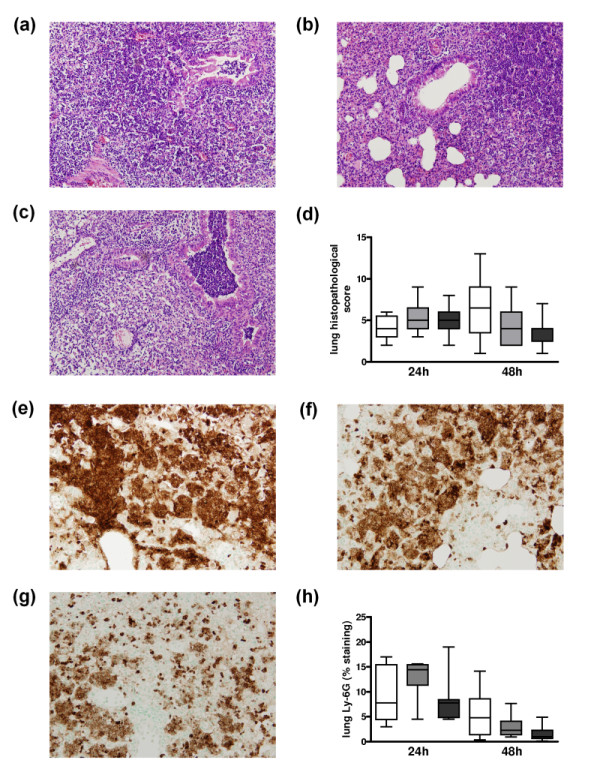
**Lung histopathology and neutrophil influx in untreated pneumococcal pneumonia**. Lung haematoxylin and eosin staining 48 hours after pneumococcal pneumonia in **(a) **wild-type mice, **(b) **mice heterozygous and **(c) **mice homozygous for the factor V Leiden mutation (original magnification × 100). **(d) **Total lung pathology score 24 and 48 hours after induction of pneumococcal pneumonia in wild-type mice (white white, *n *= 8) and mice heterozygous (light grey white, *n *= 8) or homozygous (dark grey white, *n *= 8) for the factor V Leiden mutation. Representative slides of lung Ly-6G staining (brown) 48 hours after induction of pneumococcal pneumonia in **(e) **wild-type mice, **(f) **mice heterozygous and **(g) **mice homozygous for the factor V Leiden mutation (original magnification × 100). **(h) **Quantitation of pulmonary Ly-6G content 24 and 48 hours after induction of pneumococcal pneumonia in wild-type mice (white white, *n *= 8), and mice heterozygous (light grey white, *n *= 8) or homozygous (dark grey white, *n *= 8) for the factor V Leiden mutation. Data are expressed as box-and-whisker diagrams depicting the smallest observation, lower quartile, median, upper quartile and largest observation. There were no statistical differences between the groups at either time point.

Pneumococcal pneumonia was associated with pulmonary inflammation as evidenced by the occurrence of bronchitis, interstitial inflammation, edema and endothelialitis both at 24 hours (not shown) and 48 hours after infection in all mouse strains (Figures 2a, b and 2c). There were no differences in total histopathological scores between WT, heterozygous and homozygous FVL mice at 24 hours or 48 hours after infection (Figure 2d). Moreover, there were no differences in the separate scores for bronchitis, interstitial inflammation, edema or endothelialitis (not shown). One of the prominent features in pneumococcal pneumonia is neutrophil influx into the lung parenchyma both after 24 hours (not shown) and 48 hours (Figures 2e, f and 2g). There were no differences in neutrophil influx between WT, heterozygous and homozygous FVL mice as evidenced by equal percentages of positivity in Ly-6G stainings both at 24 hours and 48 hours after infection (Figure 2h). In line with their similar histopathology and Ly-6G scores, pulmonary MPO concentrations, indicative for the number of neutrophils in lung tissue, were similar in WT, heterozygous and homozygous FVL mice at both 24 and 48 hours after infection (Table 1).

**Table 1:** Pulmonary MPO, cytokine and chemokine levels 24 and 48 hours after induction of pneumococcal pneumonia

	24 hours			48 hours		
		
	WT *n *= 8	Heterozygous *n *= 8	Homozygous *n *= 8	WT *n *= 8	Heterozygous *n *= 8	Homozygous *n *= 8
MPO (ng/mL)	5.2 (3.7-6.7)	5.0 (4.5-5.6)	4.6 (3.3-7.5)	4.6 (2.6-4.2)	3.7 (2.5-4.2)	3.4 (2.6-4.2)
TNF-α (ng/mL)	1.2 (0.8-1.8)	1.4 (0.9-2.1)	1.5 (0.7-3.0)	0.8 (0.4-1.3)	0.9 (0.5-1.5)	0.8 (0.4-1.9)
IL-6 (ng/mL)	1.5 (1.1-2.8)	1.9 (0.8-2.2)	0.5 (0.4-1.9)	1.5 (0.7-3.0)	1.5 (0.6-1.8)	0.3 (0.2-1.8)
IL-12 (pg/mL)	41 (19-53)	53 (27-102).	39 (3.1-56)	36 (18-41)	43 (23-69)	18 (3.7-38)
IFN-γ (pg/mL)	107 (57-165)	69 (56-183)	50 (35-238)	84 (39-155)	58 (47-146)	42 (24-142)
IL-10 (ng/mL)	1.1 (1.0-1.2)	0.9 (8.9-1.3)	0.8 (0.6-1.1)	1.0 (0.7-1.1)	0.9 (0.8-1.3)	0.6 (0.4-0.8)
MCP-1 (ng/mL)	8.4 (2.6-10)	10 (1.9-14)	2.8 (1.6-3.0)	6.3 (2.5-8.9)	9.0 (1.9-10)	2.6 (1.3-8.4)
KC (ng/mL)	12 (8.3-15)	12 (9.6-14)	9.5 (6.2-15)	14 (9.1-25)	8.7 (5.6-14)	10 (7.2-17)
MIP-2 (ng/mL)	16 (7.5-20)	21 (11-24)	10 (5.9-18)	13 (6.7-23)	9.6 (3.4-17)	13 (9.6-18)

Data are medians (interquartile ranges). No statistical differences between the groups at either time point.

IFN-γ, interferon-γ; KC, keratinocyte-derived chemokine; MCP-1, monocyte chemotactic protein; MIP-2, macrophage inflammatory protein-2; MPO, myeloperoxidase; WT, wild type.

To obtain further insight into the impact of the FVL mutation on pulmonary inflammation during pneumoccal pneumonia, we measured the levels of various cytokines and chemokines in lung homogenates prepared 24 and 48 hours after infection (Table 1). At both time points there were no differences in levels of TNF-α, IL-6, IL-12, IFN-γ, IL-10, MCP-1, KC or MIP-2. To obtain further insight into the impact of the FVL mutation on systemic inflammation, we measured plasma levels of the above mentioned cytokines. Plasma cytokine levels were either not different between the groups (TNF-α, IL-6, IFN-γ) or below detection (MCP-1, IL-12, IL-10) at both 24 and 48 hours after infection (data not shown).

#### Bacterial outgrowth

**Figure 3:**
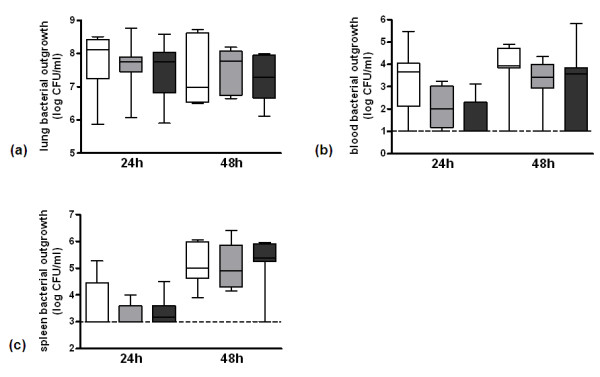
**Bacterial outgrowth in untreated pneumococcal pneumonia**. Bacterial outgrowth in **(a) **lung, **(b) **blood and **(c) **spleen 24 and 48 hours after induction of pneumococcal pneumonia in wild-type mice (white white, *n *= 8) and mice heterozygous (light grey white, *n *= 8) or homozygous (dark grey white, *n *= 8) for the factor V Leiden mutation. Data are expressed as box-and-whisker diagrams depicting the smallest observation, lower quartile, median, upper quartile and largest observation. The dotted horizontal line represents the detection limit. There were no statistical differences between the groups at either time point. CFU, colony forming units.

To investigate the effect of the FVL mutation on bacterial outgrowth and dissemination in pneumococcal pneumonia, we determined bacterial loads in lung, blood and spleen 24 and 48 hours after infection. There were no differences in bacterial loads in lung, blood or spleen between the groups at both time points (Figures 3a, b and 3c).

#### Survival

**Figure 4:**
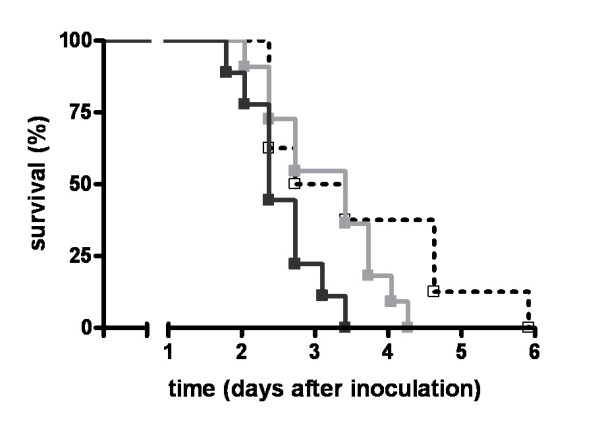
**Survival in untreated pneumococcal pneumonia**. Survival of wild-type mice (open square, dotted line white, *n *= 8) and mice heterozygous (light grey squares and line white, *n *= 11) or homozygous (dark grey squares and line white, *n *= 9) for the factor V Leiden mutation in pneumococcal pneumonia. There were no statistical differences between the groups.

To determine whether the FVL mutation impacts on mortality in pneumococcal pneumonia we performed a survival study. Despite the absence of clear differences in coagulation, fibrinolysis, pulmonary and systemic inflammation and bacterial loads, homozygous FVL mice tended to die earlier than WT and heterozygous mice (Figure 4), but this difference did not reach statistical significance (*P *= 0.09). There was no difference in survival between WT and heterozygous mice.

### Treatment with antibiotics

#### Activation of coagulation

**Figure 5:**
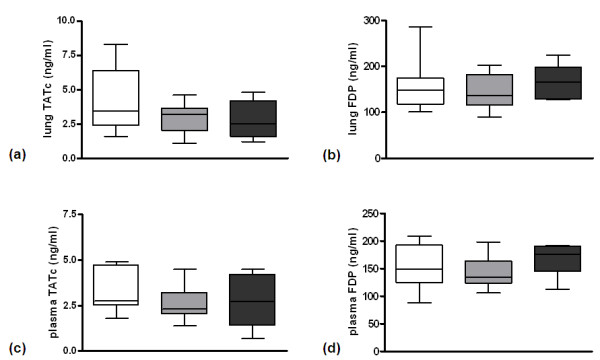
**Activation of coagulation and pulmonary fibrin deposition in antibiotic treated pneumococcal pneumonia**. Levels of **(a, c) **thrombin-antithrombin complexes (TATc) and **(b, d) **fibrin degradation products (FDP) in **(a, b) **lung and **(c, d) **plasma 48 hours after induction of pneumococcal pneumonia in wild-type mice (white white, *n *= 8) and mice heterozygous (light grey white, *n *= 8) or homozygous (dark grey white, *n *= 7) for the factor V Leiden mutation treated intraperitoneally with ceftriaxone 24 hours after infection. Data are expressed as box-and-whisker diagrams depicting the smallest observation, lower quartile, median, upper quartile and largest observation. There were no statistical differences between the groups.

To determine the impact of the FVL mutation on pneumococcal pneumonia in a clinically more relevant setting we treated mice at 24 hours of pneumonia with ceftriaxone and sacrificed them another 24 hours later. To obtain insight into local and systemic activation of coagulation in this setting we determined levels of TATc and FDP in lung homogenates (Figures 5a and 5b) and plasma (Figures 5c and 5d). There were no differences in TATc and FDP levels in lungs or plasma between WT, heterozygous and homozygous FVL mice. Also, fibrin(ogen) staining on lungs showed no differences between the groups (not shown). To determine the impact of the FVL mutation on fibrinolysis in antibiotic-treated pneumonia we determined PAI-1 levels and PAA in lungs and plasma. Again, no differences were seen between the groups (not shown).

**Figure 6:**
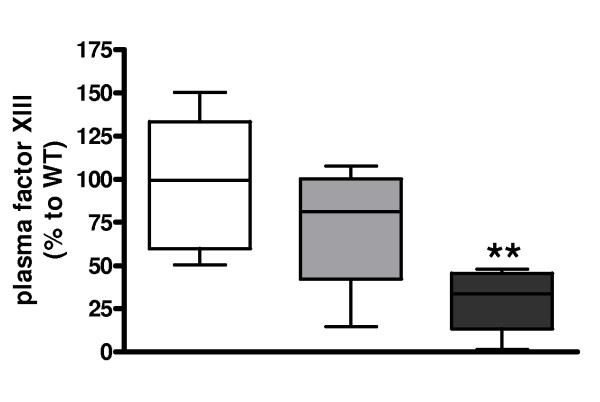
**Factor XIII depletion in antibiotic treated pneumococcal pneumonia**. Factor XIII levels 48 hours after induction of pneumococcal pneumonia in wild-type (WT) mice (white white, *n *= 8) and mice heterozygous (light grey white, *n *= 8) or homozygous (dark grey white, *n *= 7) for the factor V Leiden mutation treated intraperitoneally with ceftriaxone 24 hours after infection. Data are expressed as box-and-whisker diagrams depicting the smallest observation, lower quartile, median, upper quartile and largest observation. The mean intensity in WT mice was used as reference value (100%). ** represents statistical significance as compared with WT (*P *< 0.01, Mann Whitney U test).

To further study coagulation activation in antibiotic-treated pneumonia and a possible different phenotype of mice carrying the FVL mutation herein we performed factor XIII blotting. Remarkably, factor XIII levels were substantially lower in homozygous FVL mice as compared with WT mice (Figure 6). Heterozygous FVL mice showed a trend towards factor XIII depletion as compared with WT mice, but this was not statistically significant (*P *= 0.41).

#### Pulmonary and systemic inflammation

**Figure 7:**
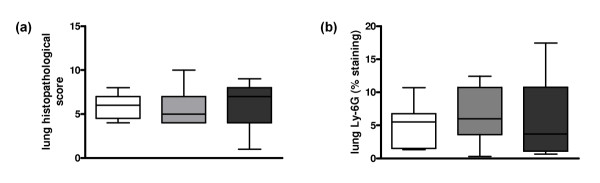
**Lung histopathology and neutrophil influx in antibiotic treated pneumococcal pneumonia**. **(a) **Total lung pathology score and **(b) **quantitation of pulmonary Ly-6G content 48 hours after induction of pneumococcal pneumonia in wild-type mice (white white, *n *= 8) and mice heterozygous (light grey white, *n *= 8) or homozygous (dark grey white, *n *= 7) for the factor V Leiden mutation treated intraperitoneally with ceftriaxone 24 hours after infection. Data are expressed as box-and-whisker diagrams depicting the smallest observation, lower quartile, median, upper quartile and largest observation. There were no statistical differences between the groups.

Total histopathology scores between ceftriaxone-treated WT, heterozygous and homozygous FVL mice were not different at 48 hours after infection (Figure 7a). Moreover, there were no differences in the separate scores for bronchitis, interstitial inflammation, edema and endothelialitis (data not shown) or in neutrophil influx, as measured by pulmonary Ly-6G staining, between WT, heterozygous and homozygous FVL mice (Figure 7b). In line with these findings, lung MPO levels were similar in WT, heterozygous and homozygous FVL mice at 48 hours after infection (Table 2).

**Table 2:** Pulmonary MPO, cytokine and chemokine levels in pneumococcal pneumonia after antibiotic treatment

	48 hours		
	
	WT *n *= 8	Heterozygous *n *= 8	Homozygous *n *= 7
MPO (ng/mL)	23 (20-24)	19 (17-21)	19 (14-21)
TNF-α (ng/mL)	0.8 (0.4-1.1)	0.6 (0.3-0.9)	0.6 (0.2-0.9)
IL-6 (ng/mL)	0.3 (0.2-0.4)	0.3 (0.2-0.5)	0.3 (0.2-0.6)
MCP-1 (ng/mL)	7.1 (5.5-9.4)	8.0 (5.3-10)	7.1 (5.6-10)
KC (ng/mL)	1.4 (1.1-1.7)	1.1 (0.9-1.3)	1.2 (0.9-1.4)

Data are medians (interquartile ranges). No statistical differences between the groups at either time point.

KC, keratinocyte-derived chemokine; MCP-1, monocyte chemotactic protein; MPO, myeloperoxidase; WT, wild type.

To obtain further insight into the impact of the FVL mutation on pulmonary inflammation during pneumoccal pneumonia treated with antibiotics, we measured the levels of various cytokines and chemokines in lung homogenates (Table 2). Lung levels of TNF-α, IL-6, MCP-1 and KC were not different between groups, whereas the concentrations of IL-12, IFN-γ, IL-10 and MIP-2 were below detection in this model. Moreover, the plasma levels of these cytokines were either similar in all three groups (TNF-α, IL-6, IFN-γ) or below detection (MCP-1, IL-12, IL-10) (data not shown).

#### Bacterial outgrowth

To investigate the effect of the FVL mutation on bacterial outgrowth in pneumococcal pneumonia in the context of antibiotic therapy, we determined bacterial loads in lung, blood and spleen. As expected, pulmonary bacterial loads were lower than in the non-treated setting. There were no differences in bacterial loads in the lungs between the groups (data not shown). Cultures of blood and spleen remained sterile.

#### Survival

**Figure 8:**
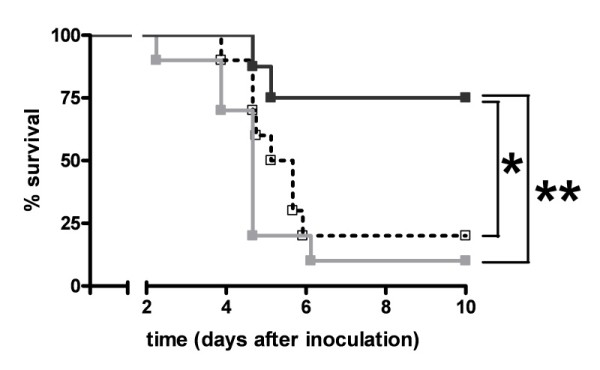
**Survival in antibiotic treated pneumococcal pneumonia**. Survival of wild-type mice (open square, dotted line white, *n *= 10) and mice heterozygous (light grey squares and line white, *n *= 10) or homozygous (dark grey squares and line white, *n *= 8) for the factor V Leiden mutation treated intraperitoneally with ceftriaxone 24 hours after induction of pneumococcal pneumonia. * indicates statistical significance for the comparison between homozygous factor V Leiden and wild-type mice (*P *< 0.05 log rank test), ** indicates statistical significance for the comparison between homozygous and heterozygous factor V Leiden mice (*P *< 0.01 log rank test).

To determine whether the FVL mutation impacts on survival during pneumococcal pneumonia in the context of antibiotic therapy we performed a survival study. Remarkably, although most WT and heterozygous FVL mice died between day two and six, most homozygous FVL mice were rescued by ceftriaxone treatment (Figure 8). Survival between WT and heterozygous mice was not different.

## Discussion

*S. pneumoniae *is the leading causative pathogen in CAP and a major cause of morbidity and mortality in humans. Local pulmonary as well as systemic activation of coagulation and downregulation of anticoagulant mechanisms and fibrinolysis have been shown in preclinical models of and patients with pneumococcal pneumonia and sepsis [6-10]. The high prevalence of the FVL mutation, despite its prothrombotic effects [12], suggests that the mutation might be subject to positive selection pressure during evolution [13]. Indeed, some data indicate that heterozygous FVL carriers may have a survival benefit in severe sepsis, although that conclusion has been disputed [16,17]. We here show in murine models of untreated and antibiotic-treated pneumococcal pneumonia that heterozygosity or homozygosity for the FVL mutation do not consistently alter inflammation, bacterial outgrowth and activation of coagulation and fibrinolysis in the lungs or plasma except for coagulation factor XIII levels: Homozygous FVL mice display evidence of coagulation factor XIII depletion at 48 hours in the context of antibiotic treatment and are protected against mortality in this setting, but not in an untreated setting, when compared with WT and heterozygous FVL mice.

The FVL mutation results in resistance of FVa to inactivation by APC [11], leading to increased thrombin generation, which presumably accounts for the elevated risk of thrombotic events in FVL carriers [28]. Therefore, in theory, the FVL mutation might result in further aggravation of the procoagulant state that commonly accompanies pneumococcal pneumonia and sepsis [6-10]. However, we were unable to demonstrate an altered local or systemic procoagulant response in heterozygous or homozygous FVL mice in *S. pneumoniae *pneumonia with or without antibiotic treatment, as evidenced by unchanged lung and plasma TATc and FDP levels and unaltered fibrin deposition in lungs 24 hours after infection. In addition, fibrinolysis was unaltered in FVL mice, as reflected by comparable levels of PAI-1 and PAA. Notably, 48 hours after infection not treated with antibiotics we found slightly lower TATc levels in the lungs of heterozygous FVL mice as compared with WT mice and mice homozygous for the FVL mutation. This unexpected finding was not accompanied by changes in lung FDP levels, an effect on pulmonary fibrin deposition or altered systemic coagulation activation as measured by plasma TATc and FDP levels. Also, in mice treated with ceftriaxone, local and systemic levels of TATc and FDP were not different between the groups. As the FVL mutation has been associated with APC resistance in both humans [11] and mice [20], it is difficult to envision how heterozygosity for the FVL mutation would lead to lower pulmonary TATc levels, especially when considering that at this time point homozygosity for the FVL mutation did not influence lung TATc levels. As such, a clear explanation for this difference amid a whole series of similar coagulation, fibrinolysis and inflammation markers is lacking. Our current data, indicating that the FVL mutation does not consistently influence the global procoagulant response in untreated and antibiotic-treated pneumococcal pneumonia, are in accordance with previous studies that reported similar plasma concentrations of biomarkers of coagulation activation in patients with or without the FVL mutation in severe sepsis [16] and similar rises in plasma and peritoneal TATc levels in FVL mice and WT mice injected intraperitoneally with *E. coli *[18].

It has been suggested that the FVL mutation may impact on acute inflammatory responses in the lung [29]. The current study does not support this notion: relative to WT animals, heterozygous and homozygous FVL mice showed an unaltered inflammatory response in their lungs upon infection with *S. pneumoniae*, as reflected by similar histopathology scores of lung tissue, a similar influx of neutrophils to the site of infection and similar cytokine and chemokine concentrations in lung homogenates, both in the presence or absence of antibiotic treatment. Moreover, plasma cytokine levels were unaltered in FVL mice in both settings. In accordance, the FVL mutation did not influence baseline plasma IL-6 levels in patients with severe sepsis [16] and the induction of systemic cytokine release during Gram-negative sepsis did not differ between FVL and WT mice [18].

In our study, local as well as systemic bacterial outgrowth was unaltered in heterozygous and homozygous FVL mice as compared with WT animals in both untreated and antibiotic-treated pneumococcal pneumonia. These data are in correspondence with a preclinical model of severe Gram-negative sepsis in which bacterial outgrowth did not differ between WT mice and heterozygous and homozygous FVL carriers [18].

A remarkable finding in our study was the clear survival benefit of homozygous FVL mice in pneumococcal pneumonia treated with antibiotics, a protective effect that was not observed in untreated respiratory tract infection by *S. pneumoniae*. Although a possible beneficial effect of the FVL mutation on infectious disease survival during evolution should be investigated in the absence of antibiotic interventions, studying the impact of the FVL mutation on the outcome of pneumococcal pneumonia in the context of antibiotic treatment is more relevant from the clinical perspective of today's health care. In accordance with earlier studies not using antibiotic treatment and showing an unaltered mortality of FVL mice in experimental Gram-negative [18] and Gram-positive sepsis [19], our investigation did not reveal different mortality rates in FVL and WT mice not treated with ceftriaxone. To our knowledge we are the first to investigate the effect of the FVL mutation in a preclinical model of severe infection in which animals were treated with antibiotics. In this setting, homozygous FVL mice were almost completely rescued by antibiotic treatment, whereas the vast majority of heterozygous FVL and WT mice died. Interestingly, our data bear some resemblance with earlier findings reported in patients with severe sepsis, who obviously were all treated with antibiotics [16]. Indeed, heterozygous FVL carriers with severe sepsis had a reduced mortality as compared with non-carriers (13.9% versus 27.9%), whereas the number of homozygous FVL carriers was too low to study the impact on survival [16]. As in our animal study, human FVL carriers displayed unaltered procoagulant and inflammatory responses during severe sepsis [16].

As FXIII, the main crosslinker of fibrin, has been linked to organ failure in septic shock in rabbits [30] we performed FXIII blots in plasma of antibiotic-treated mice. Remarkably, FXIII was depleted in homozygous FVL mice as compared with WT and heterozygous FVL mice. Although our data can not establish whether FXIII really plays a pathophysiological role in our model or is merely a read out of disease severity, it is striking that factor XIII was the only parameter among many in which we found a difference between the protected homozygous FVL mice on the one hand and WT and heterozygous FVL mice on the other hand before mortality occurred. More research is warranted to investigate the mechanisms by which the FVL mutation impacts on survival in clinical sepsis [16] and murine pneumococcal pneumonia (the current study) in the context of antibiotic therapy.

## Conclusions

Homozygosity for the FVL mutation was associated with a survival benefit in antibiotic treated pneumococcal pneumonia, without influencing the procoagulant or inflammatory response. These findings resemble earlier reports in heterozygous human FVL carriers with severe sepsis. Homozygosity for the FVL mutation was associated with lower levels of FXIII in antibiotic-treated pneumococcal pneumonia, a finding which requires further study.

## Key messages

• FVL does not alter coagulation activation in untreated and antibiotic-treated pneumococcal pneumonia except for FXIII levels in antibiotic-treated pneumonia.

• FVL does not alter inflammation or bacterial outgrowth in untreated and antibiotic-treated pneumococcal pneumonia.

• FVL does not alter survival in untreated pneumococcal pneumonia.

• Homozygosity for FVL protects against lethality in antibiotic treated pneumococcal pneumonia, which is possibly related to a difference in factor XIII depletion.

## Abbreviations

APC: activated protein C; BSA: bovine serum albumin; CAP: community-acquired pneumonia; CFU: colony forming units; ELISA: enzyme-linked immunosorbent assay; FDP: fibrin degradation products; FVA: activated factor V; FVL: factor V Leiden; H&E: hematoxylin & eosin; IFN-γ: interferon-γ; IG: immunoglobulin; IL: interleukin; KC: keratinocyte-derived chemokine; MCP-1: monocyte chemoattractant protein-1; MIP-2: macrophage inflammatory protein-2; MPO: myeloperoxidase; PAA: plasminogen activator activity; PAI-1: plasminogen activator inhibitor-1; TATC: thrombin-antithrombin complexes; TNF-α: tumor necrosis factor-α; WT: wild-type.

## Competing interests

The authors declare that they have no competing interests.

## Authors' contributions

MS participated in the design of the study, carried out the *in vivo *experiments and drafted the manuscript. CV participated in the design of the study and helped to draft the manuscript. JJR performed pathology scoring, prepared part of the figures and helped to draft the manuscript. ML performed coagulation measurements and helped to draft the manuscript. TP participated in the design of the study, supervised the study and helped to draft the manuscript. All authors read and approved the manuscript.
